# Rigid TiO_2−*x*_ coated mesoporous hollow Si nanospheres with high structure stability for lithium-ion battery anodes†

**DOI:** 10.1039/c8ra01661e

**Published:** 2018-04-23

**Authors:** Yongli Yu, Gang Li, Xu Chen, Weiguo Lin, Junfeng Rong, Wensheng Yang

**Affiliations:** State Key Laboratory of Chemical Resource Engineering, Beijing University of Chemical Technology Beijing 100029 P. R. China yangws@mail.buct.edu.cn; Research Institute of Petroleum Processing, Sinopec Beijing 100083 P. R. China rongjf.ripp@sinopec.com

## Abstract

Rigid oxygen-deficient TiO_2−*x*_ coated mesoporous hollow Si nanospheres with a mechanically and electrically robust structure have been constructed through a facile method for high-performance Li-ion battery anodes. The mesoporous hollow structure provides enough inner void space for the expansion of Si. The oxygen-deficient TiO_2−*x*_ coating has functions in three aspects: (1) avoiding direct contact between Si and the electrolyte; (2) suppressing the outward expansion of the mesoporous hollow Si nanospheres; (3) improving the conductivity of the composite. The combined effect leads to high interfacial stability and structural integrity of both the material nanoparticles and the whole electrode. By virtue of the rational design, the composite yields a high reversible specific capacity of 1750.4 mA h g^−1^ at 0.2 A g^−1^, an excellent cycling stability of 1303.1 mA h g^−1^ at 2 A g^−1^ with 84.5% capacity retention after 500 cycles, and a high rate capability of 907.6 mA h g^−1^ even at 4 A g^−1^.

## Abstract 2

A conductive TiO_2−*x*_ shell suppresses the outward expansion of Si to maintain high interfacial stability and structural integrity.

## Graphical abstract

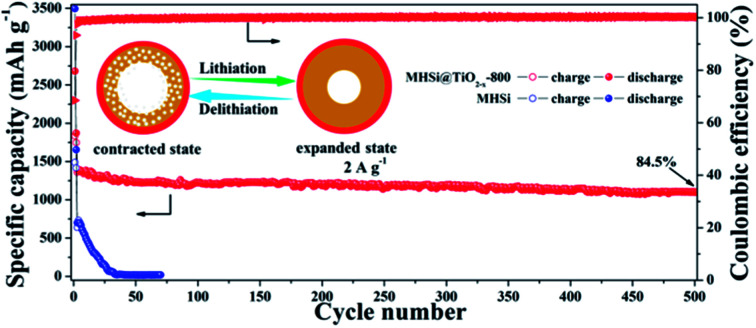

## Introduction

The fast development of electric vehicles requires rechargeable Li-ion batteries (LIBs) with high-energy density, long-cycle life, and high-rate performances.^1–4^ As key components, electrode materials dominate the primary electrochemical properties of LIBs. Owing to the low theoretical specific capacity (372 mA h g^−1^) of graphite, which is the primary anode material for LIBs in current commercial applications, many electrochemically active materials with high specific capacities have been developed.^5–8^ Silicon (Si) is proposed as one of the most promising next-generation anode materials owing to its high theoretical specific capacity (3578 mA h g^−1^), natural abundance, and environmental benignity.^9–11^ However, the inherently low electrical conductivity of Si limits its transport kinetics at high current densities. Besides, the large volume change (∼300%) during the lithiation/delithiation processes leads to pulverization of Si, fracture of the electrode, and an unstable solid electrolyte interphase (SEI), seriously restricting the practical application of Si anodes.^12–14^

To circumvent these challenges, two main strategies are proposed. The first strategy is to design and optimize the structure of Si. Various structures of Si, such as nanoparticles,^15^ nanomembranes,^16^ nanowires,^17^ nanotubes,^18^ hollow nanospheres,^19^ and porous structures,^20–23^ have been designed and reported. Particularly, hollow Si nanospheres with large inner void space and thin shell can accommodate the stress induced by the volume change and decrease the diffusion distance of the electrolyte and Li^+^ during cycling.^24^ However, the high specific surface area and the direct contact between the surface of hollow Si nanospheres and the electrolyte will lead to more Li^+^ consumption and an unstable SEI growth. In addition, the chemical vapor deposition (CVD) process which uses the hard sacrificial templates and toxic SiH_4_ is the most used method to prepare hollow Si nanospheres.^19,25,26^ However, the CVD method is quite complicated and dangerous for large scale production.^26^ Although partial magnesiothermic reduction of solid silica spheres has been reported to synthesize hollow Si nanosphere,^27^ it is still challenging to synthesize porous hollow Si nanospheres with well controlled morphology and mesoporosity. Another strategy is to combine silicon with other electronically conductive materials, such as Si/carbon composites and Si/metal composites.^28–31^ Carbon material coatings are usually used to improve the electrical conductivity of Si and to provide a barrier layer to isolate Si active materials from the electrolytes.^32,33^ However, the carbon shell in close contact with the Si core is prone to fracture due to the low strength of amorphous carbon and the large volume expansion of Si.^34^ More miserably, during the carbonation processes of the carbon coatings, other by-products may sometimes generate, for example, electrochemically inert SiC.^35,36^ As an alternative, titanium oxide (TiO_2_) has got a lot of attention because it shows good structural stability with negligible volume change.^37–40^ More excitingly, Cui and his cooperators have shown that TiO_2_ shell exhibits 5× greater strength than an amorphous carbon shell.^41^ Specially, oxygen-deficient TiO_2−*x*_ not only shows the good structural stability of TiO_2_, but also yields high electrical conductivity due to its narrower band gap.^42–44^ For example, a core–shell structured microfiber with Si nanoparticles core and TiO_2−*x*_/C shell has been reported and showed enhanced electrochemical performance.^45^

In this work, we developed a facile approach for the fabrication of rigid TiO_2−*x*_ coated mesoporous hollow Si nanospheres (denoted as MHSi@TiO_2−*x*_) through the magnesiothermic reduction of hollow silica nanospheres (HSiO_2_) to generate mesoporous hollow silicon nanospheres (MHSi), tetrabutyl titanate (TBOT) hydrolysis on the MHSi surface, and sequent calcination in an inert atmosphere. The mesoporous hollow structure provides enough inner void space for the expansion of Si. The TiO_2−*x*_ coating not only avoids the direct contact between Si and the electrolyte, but also effectively confines the outward expansion of Si nanospheres. The core–shell combined effect leads to high interfacial stability and structural integrity of both the material nanoparticles and the whole electrode. Additionally, the conductive TiO_2−*x*_ coating tremendously increases the electrical conductivity of the composite. The rationally designed composite therefore exhibited remarkable lithium storage properties as an anode for LIBs, such as improved large reversible specific capacity, excellent long-term cycling stability, and high rate capability, compared with the pure MHSi.

## Experimental

### Synthesis of hollow SiO_2_ spheres (HSiO_2_)

The synthesis of HSiO_2_ was based on the method reported in the literature.^46^ The concentrated ammonia aqueous solution (4 mL, 28 wt%) was added into an ethanol aqueous solution (120 mL absolute ethanol and 200 mL de-ionized water). Then, the cetyltrimethylammonium bromide (CTAB) (0.6 g) was also added into the ethanol aqueous solution and sonicated in a sonicator bath for 15 min. The tetraethoxysilane (TEOS) (4 mL) was dropwise added to the above solution under stirring, then, the solution was stirred (500 rpm) at 30 °C for 24 h. The white precipitation was collected by centrifugation and incubated in de-ionized water (400 mL) at 90 °C. After 24 h, the precipitation was collected by centrifugation and washed with de-ionized water. Then the obtained white precipitation was dispersed again into an ethanol solution (320 mL) containing HCl (900 μL, 37%) and stirred (500 rpm) at 60 °C for 3 h to remove CTAB. The hollow SiO_2_ sphere (HSiO_2_) was obtained.

### Synthesis of mesoporous hollow Si spheres (MHSi)

The transformation from HSiO_2_ to MHSi was realized by magnesiothermic reduction. HSiO_2_ (1 g), magnesium powder (0.8 g, 200–300 mesh) and NaCl (10 g) were fully mixed by grinding for 30 min. Then, the mixture was placed in a sealed stainless steel tube filled with Ar atmosphere and heated in a tube furnace under Ar flow at 680 °C for 4 h with a ramp rate of 3 °C min^−1^. The obtained brown powder was soaked in a 1 mol L^−1^ HCl aqueous solution for 5 h to remove the MgO and MgSi_2_. Then the precipitation was collected by centrifugation and etched by 5% HF for 30 min to remove the residual silica. The brown product was collected by centrifugation and dried under vacuum at 60 °C for 5 h. The mesoporous hollow Si spheres (MHSi) was obtained.

### Synthesis of TiO_2−*x*_ coated mesoporous hollow Si spheres (MHSi@TiO_2−*x*_)

The concentrated ammonia solution (0.3 mL, 28 wt%) was added into the absolute ethanol (100 mL), then the MHSi (100 mg) were dispersed in the above solution under sonication for 15 min. Afterward, the tetrabutyl titanate (TBOT) (0.45 mL) was also added dropwise in 10 min. The reaction proceeded under continuous stirring (400 rpm) at 45 °C for 12 h. The precipitation was collected by centrifugation and washed with deionized water and ethanol for 3 times, respectively. Then, the obtained precipitation was dried under vacuum at 100 °C for 6 h. Finally, the precipitation was placed in a corundum crucible and heated in a tube furnace under Ar flow at 500 °C, 600 °C, 700 °C, 800 °C and 900 °C, respectively, for 4 h with a ramp rate of 3 °C min^−1^. The products were denoted as MHSi@TiO_2−*x*_-500, MHSi@TiO_2−*x*_-600, MHSi@TiO_2−*x*_-700, MHSi@TiO_2−*x*_-800 and MHSi@TiO_2−*x*_-900, respectively.

### Synthesis of TiO_2−*x*_ and TiO_2_

The concentrated ammonia solution (0.3 mL, 28 wt%) was added into the absolute ethanol (100 mL), then the TBOT (0.45 mL) was added dropwise into the above solution in 10 min, and the reaction proceeded under continuous stirring (400 rpm) at 45 °C for 12 h. The precipitation was collected by centrifugation and washed with deionized water and ethanol for 3 times, respectively. Then, the obtained precipitation was dried under vacuum at 100 °C for 6 h. Finally, the precipitation was placed in a corundum crucible and heated in a tube furnace under Ar flow at 800 °C for 4 h with a ramp rate of 3 °C min^−1^, generating TiO_2−*x*_. The TiO_2_ was synthesized through the same process except calcination in air at 800 °C.

### Material characterization

The morphologies of the obtained materials were characterized using a ZEISS Supra 55 scanning electron microscopy (SEM) at a voltage of 20 kV and a JEOL JEM-3010 high-resolution transmission electron microscopy (HRTEM) at an accelerating voltage of 200 kV. The compositions and structures of the materials were confirmed from X-ray diffraction (XRD) patterns which were recorded on a D/max-Ultima III diffractometer with Cu K_α_ radiation (*λ* = 0.154 nm) and X-ray photoelectron spectroscopy (XPS) spectra which were performed on a Thermo ESCALAB250 instrument with an internal standard C 1s peak at 284.8 eV. Brunauer–Emmett–Teller (BET) surface area and the corresponding pore size distributions of the materials were obtained from the analysis of the N_2_ adsorption/desorption isotherms which were performed on a Micromeritics ASAP 2010 instrument.

### Electrochemical measurements

CR2032 coin-type cell, in which the Li metal was both the counter and the reference electrode, was used to evaluate the electrochemical properties of the materials. The working electrodes were prepared through multiple programs. The active material, acetylene black, sodium carboxymethyl cellulose (CMC) and butadiene styrene rubber (SBR) with the mass ratio of 70 : 10 : 10 : 10 were mixed into a homogeneous slurry through grinding and stirring. Then the obtained slurry was deposited on the Cu foil and dried at 100 °C in vacuum for 12 h to generate the working electrodes. The CR2032 coin-type cell was assembled in an Ar-filled glove box. In the cell, the electrolyte was composed of 1 mol L^−1^ LiPF_6_ dissolving in the ethylene carbonate (EC), dimethyl carbonate (DC), and ethyl methyl carbonate (EMC) with the volume ratio of 1 : 1 : 1. Fluoroethylene carbonate (FEC) was added into the electrolyte as the electrolyte additive, and the content of FEC in the electrolyte was 10%. Cyclic voltammetry (CV) was recorded on a CHI660E electrochemical workstation at a scan rate of 0.1 mV s^−1^ in the potential range of 1.5–0.01 V *vs.* Li/Li^+^. galvanostatic discharge/charge measurements were performed on a CT2001A-LAND test system at room temperature with the potential range of 0.01–1.2 V *vs.* Li/Li^+^. Electrochemical impedance spectra (EIS) was obtained from an electrochemical IM6e impedance analyzer in the frequency range of 100 000–0.01 Hz with a perturbation voltage of 0.005 V. The specific capacities of the materials and the current densities were calculated based on the active materials, and the mass loading of the active material was about 0.65–0.7 mg cm^−2^.

## Results and discussion

**Fig. 1:**
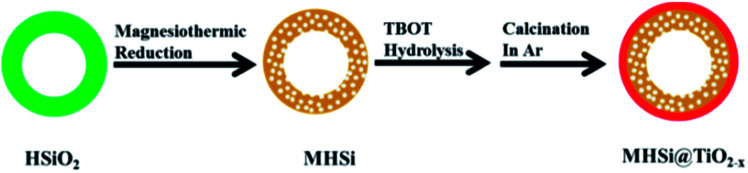
Schematic illustration for the synthesis of MHSi@TiO_2−*x*_ composite.

The preparation procedure for the MHSi@TiO_2−*x*_ composite is illustrated in Fig. 1. Firstly, uniform HSiO_2_ nanospheres were synthesized *via* a spontaneous self-transformation method by aging the solid silica spheres in water. Secondly, the MHSi spheres were obtained through magnesiothermic reduction of HSiO_2_. Lastly, a uniform TiO_2−*x*_ coating on the MHSi was realized by the hydrolysis of TBOT and the subsequent calcination process, generating the MHSi@TiO_2−*x*_ composite.

**Fig. 2:**
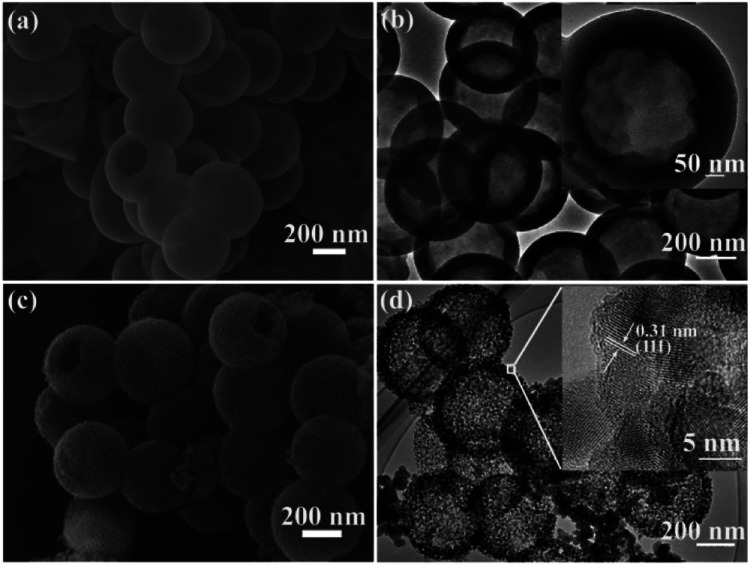
(a) SEM image and (b) HRTEM image of HSiO_2_, (c) SEM image and (d) HRTEM image of MHSi.

As shown in Fig. 2a, SEM was employed to investigate the morphology of HSiO_2_. The HSiO_2_ is spherical and uniform with an average diameter of approximately 500 nm, and a hollow structure can be clearly observed from a broken sphere. The HRTEM image (Fig. 2b) demonstrates that HSiO_2_ has a hollow structure with a wall thickness of about 80 nm (embedded figure in Fig. 2b). The formation of the hollow structure in HSiO_2_ is attributed to the different compact degree of SiO_2_ spheres between their outer layer and inner section, and the inner section is more easily etched by water molecules.^46^ After magnesiothermic reduction, the pristine smooth surface gets rough and mesoporous (Fig. 2c), but the hollow structure is still maintained (Fig. 2d). As shown in the embedded figure in Fig. 2d, the shell of MHSi sphere is composed of silicon crystallites of 10–20 nm in diameter and the measured interplanar distance of 0.31 nm is ascribed to the (111) plane of the cubic Si.

The MHSi@TiO_2−*x*_ composite was prepared *via* the hydrolysis of TBOT on the surface of MHSi and the subsequent calcination process. Compared with the white TiO_2_ (Fig. S1†) and the black TiO_2−*x*_ (Fig. S2†), the MHSi@TiO_2−*x*_ composite (Fig. S3†), especially the samples obtained at 700 °C, 800 °C, and 900 °C, showed dark-green color, implying the presence of nonstoichiometric TiO_2−*x*_, because many reports have shown that the coloration of TiO_2_ is in relation with the electronic structure.^42,43,47^ Considering the sample obtained at 800 °C (designated as MHSi@TiO_2−*x*_-800) showed the best electrochemical properties (Fig. S4 and the corresponding detailed discussion displayed in the ESI†), the composition, structure, and structural changes after lithiation and delithiation of MHSi@TiO_2−*x*_-800 were further studied in more detail.

**Fig. 3:**
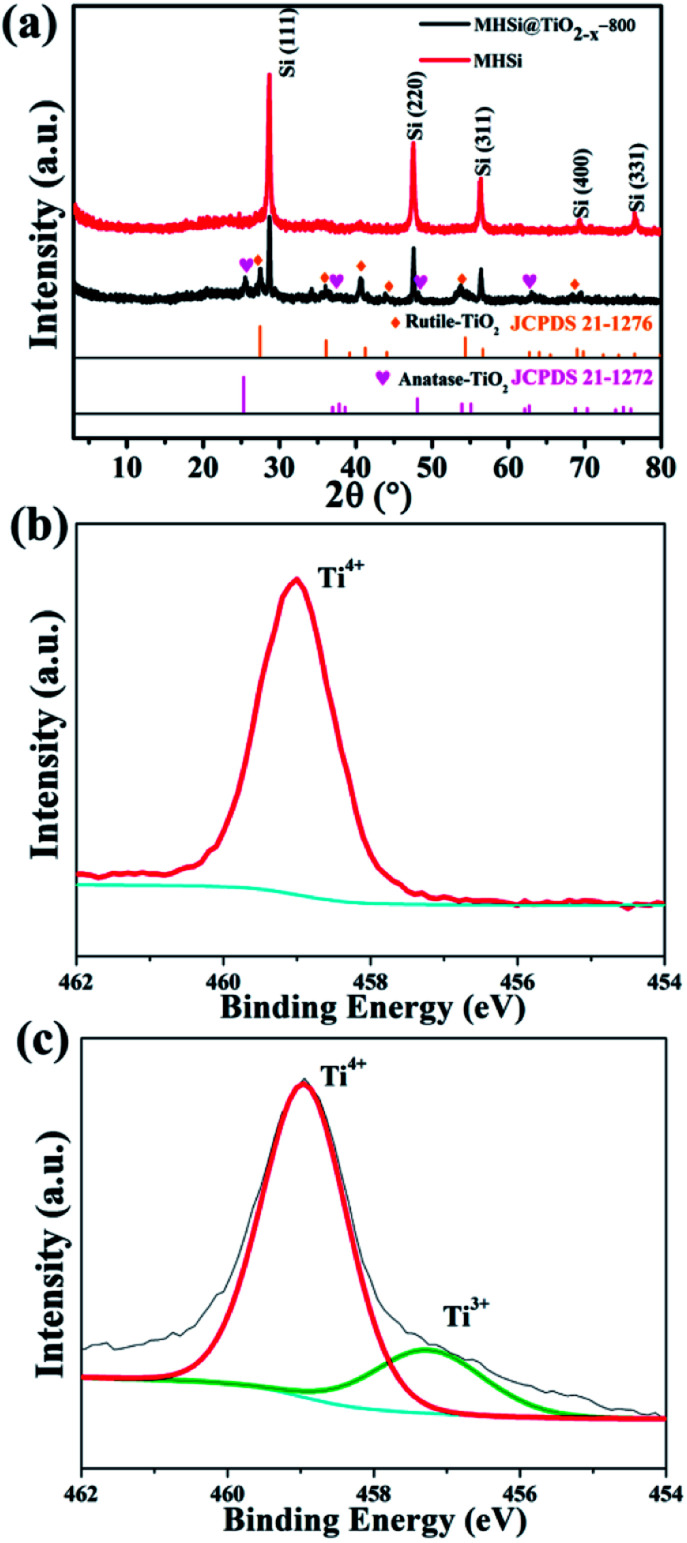
(a) XRD patterns of MHSi and MHSi@TiO_2−*x*_-800, Ti 2p XPS spectra of (b) TiO_2_ and (c) MHSi@TiO_2−*x*_-800.

The composition of MHSi@TiO_2−*x*_-800 was confirmed using X-ray diffraction (XRD). The sharp peaks at 28.5°, 47.4°, 56.2°, 69.3°, and 76.5° in the XRD patterns of MHSi and MHSi@TiO_2−*x*_-800 in Fig. 3a correspond to cubic Si (JCPDS 27-1402). The peaks signed by brown rhombus in the XRD pattern of MHSi@TiO_2−*x*_-800 correspond to rutile TiO_2_ (JCPDS 21-1276), and the peaks signed by pink peach heart correspond to anatase TiO_2_ (JCPDS 21-1272), indicating that the TiO_2−*x*_ in MHSi@TiO_2−*x*_-800 is composed of rutile TiO_2_ and anatase TiO_2_. X-ray photoelectron spectroscopy (XPS) was used to verify the presence of nonstoichiometric TiO_2_ (Fig. 3b and c). Although both white TiO_2_ and dark-green MHSi@TiO_2−*x*_-800 show typical Ti 2p XPS spectra with Ti^4+^ characteristics (Ti 2p_3/2_ peak at 459.0 eV of binding energy), MHSi@TiO_2−*x*_-800 also exhibits a shoulder peak near 457.4 eV, which is the characteristic of Ti^3+^.^45,48^ The ratio of Ti^4+^ to Ti^3+^ is calculated to be 87.7/12.3, so the TiO_2−*x*_ in MHSi@TiO_2−*x*_-800 is assumed to be TiO_1.939_. According to the analysis of energy dispersive spectrometer (EDS) spectrum of MHSi@TiO_2−*x*_-800, the content of TiO_2−*x*_ in MHSi@TiO_2−*x*_-800 is 27.6% (Fig. S6† and the corresponding discussion).

**Fig. 4:**
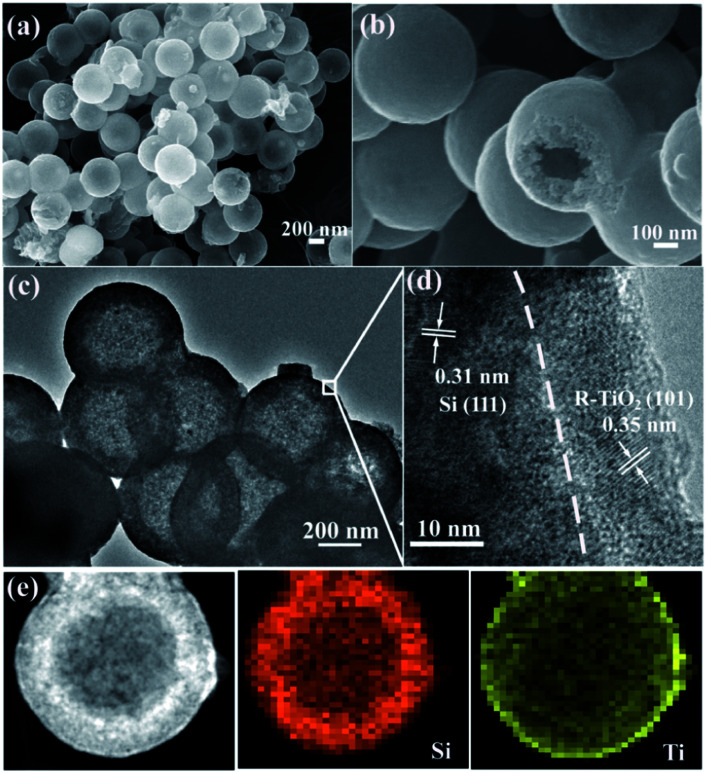
(a and b) SEM images and (c and d) HRTEM images of MHSi@TiO_2−*x*_-800, (e) HRTEM image and energy-dispersive X-ray elemental mapping of Si and Ti of MHSi@TiO_2−*x*_-800.

As shown in Fig. 4a and b, the morphology of MHSi@TiO_2−*x*_-800 was characterized using SEM. The uniform spheres with smooth surface are clearly observed. The hollow structures, observed from the broken spheres, are still maintained. HRTEM was also used to investigate the composition and morphology of MHSi@TiO_2−*x*_-800 in more depth. The mesoporous hollow structure can be clearly observed from Fig. 4c. A closer view of the composite is shown in Fig. 4d. The crystalline lattice of 0.31 nm in the inner layer corresponds to the (111) plane of the Si crystal, and that of 0.35 nm in the outer layer corresponds to the (101) plane of the rutile TiO_2_ crystal. Combined with the XRD and XPS analysis, the outer layer is TiO_2−*x*_. As shown in Fig. 4e, a HRTEM image, together with energy-dispersive X-ray elemental mapping of Si and Ti, clearly reveals that the mesoporous hollow Si nanospheres are situated in the endothecium of the composite and capsulated by a uniform TiO_2−*x*_ shell. The thickness of the Si layer is about 80 nm, and that of the TiO_2−*x*_ shell is 15–20 nm.

**Fig. 5:**
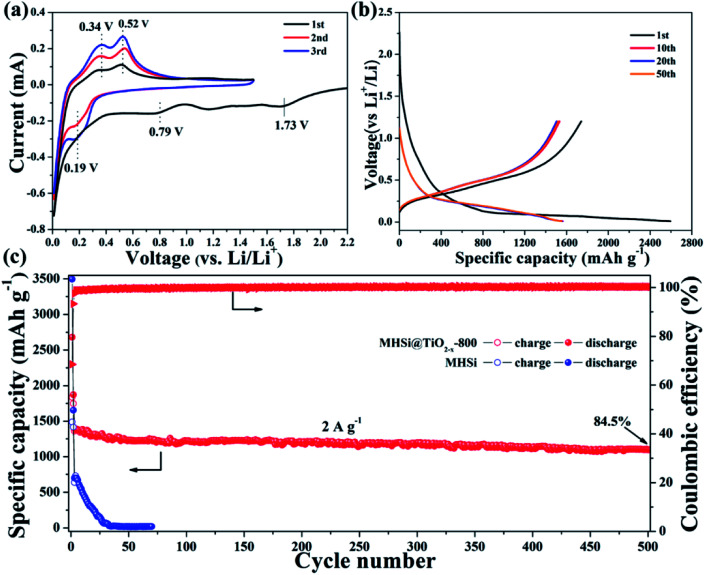
(a) CV curves of MHSi@TiO_2−*x*_-800 measured at a scan rate of 0.1 mV s^−1^ within 0.01–1.5 V *vs.* Li/Li^+^. (b) Discharge/charge profiles of MHSi@TiO_2−*x*_-800 at 0.4 A g^−1^. (c) Cyclic performances of MHSi and MHSi@TiO_2−*x*_-800 at 2 A g^−1^ (the first two cycles were performed at a current density of 0.2 A g^−1^).

The electrochemical properties of the MHSi@TiO_2−*x*_-800 composite were further evaluated based on CV and galvanostatic discharge/charge measurements. Fig. 5a shows the CV curves of the MHSi@TiO_2−*x*_-800 composite. In the first discharge process, the small peak recorded at 1.73 V is ascribed to the lithiation of the TiO_2−*x*_ shell, and that observed at 0.79 V, which disappears from the second cycle, is attributed to the formation of a solid electrolyte interface (SEI). A large peak at ∼0 V can be observed in the first discharge process, and a clear shoulder peak also appears at 0.19 V in the subsequent cycles; this is related to the lithiation of crystalline Si to form amorphous Li_*x*_Si alloys.^49^ In the first charge process, two clear peaks observed at 0.34 and 0.52 V, respectively, are related to the delithiation of Li_*x*_Si to amorphous Si. During the subsequent cycles, the peaks remain their positions, but their intensities increase, indicating that the lithiation/delithiation processes are highly reversible and the electrode is activated gradually. The charge/discharge profiles of the MHSi@TiO_2−*x*_-800 composite at the current density of 0.4 A g^−1^ are shown in Fig. 5b. The composite delivers a high initial discharge specific capacity of 2674.6 mA h g^−1^. The initial coulombic efficiency is 66.2% because of the formation of an SEI layer and the Li^+^ consumed by TiO_2−*x*_. The first discharge profile is different from the others because of the lithiation of crystalline Si to amorphous Li_*x*_Si. The charge profile of the 10th cycle almost overlaps with those of the 20th and the 50th cycle, revealing high cycling stability. Excitingly, the MHSi@TiO_2−*x*_-800 composite exhibits an outstanding cyclic performance (Fig. 5c). At a current density of 2 A g^−1^, MHSi@TiO_2−*x*_-800 delivers a high reversible specific capacity of 1303.1 mA h g^−1^ and 84.5% capacity retention after 500 cycles. According to the reversible specific capacity (Fig. S4†) and the content (Fig. S6† and the corresponding discussion) of TiO_2−*x*_, the contribution of TiO_2−*x*_ to the total specific capacity of MHSi@TiO_2−*x*_-800 is only 38.6 mA h g^−1^. In addition, the coulombic efficiency after 10 cycles maintains higher than 99%, revealing the good interface stability during cycling. In contrast, the initial coulombic efficiency of MHSi is only 40.3%, which is ascribed to the large specific surface area (Fig. S7†) and the direct contact with the electrolyte. Although MHSi delivers a high initial charge specific capacity, the specific capacity fades fast in the subsequent cycles, indicating that the TiO_2−*x*_ shell plays a key effect on the cycling stability.

**Fig. 6:**
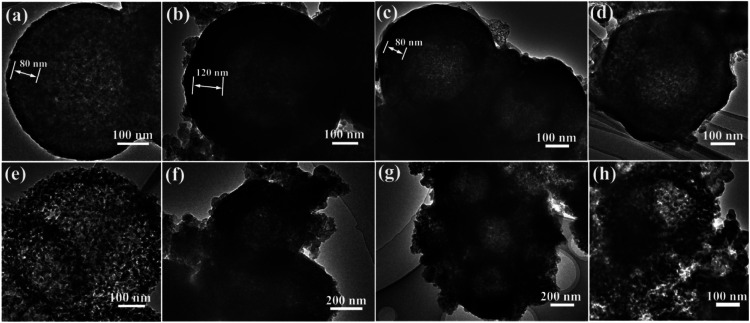
HRTEM images of (a) the pristine MHSi@TiO_2−*x*_-800, (b) MHSi@TiO_2−*x*_-800 after the first lithiation (discharge to 0.01 V), (c) MHSi@TiO_2−*x*_-800 after the first delithiation (charge to 1.2 V), (d) MHSi@TiO_2−*x*_-800 after 50 cycles, (e) the pristine MHSi, (f) MHSi after the first lithiation (discharge to 0.01 V), (g) MHSi after the first delithiation (charge to 1.2 V), and (h) MHSi after 50 cycles.

**Fig. 7:**
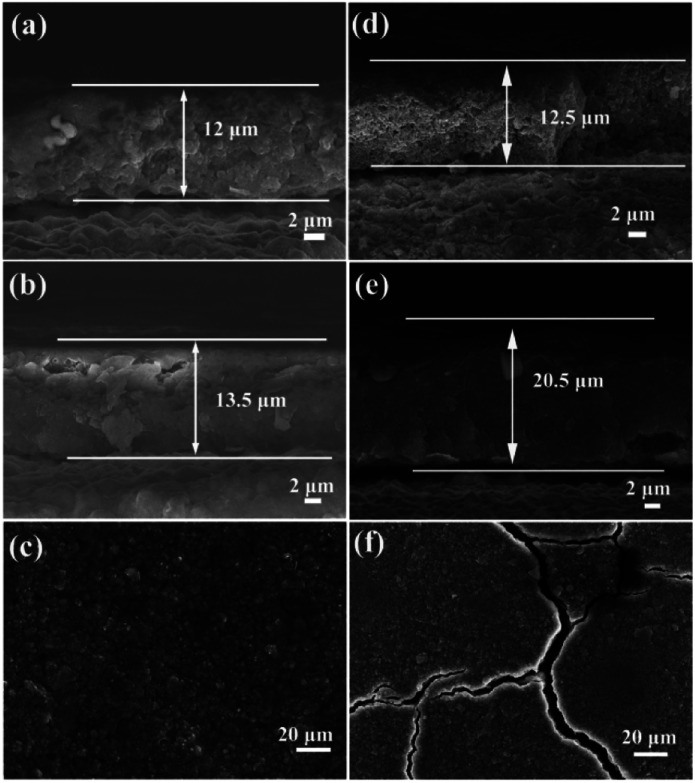
Cross-sectional SEM images of MHSi@TiO_2−*x*_-800 electrode (a) before cycling and (b) after the first lithiation (discharge to 0.01 V). (c) Top view SEM image of MHSi@TiO_2−*x*_-800 electrode after 500 cycles. Cross-sectional SEM images of MHSi electrode (d) before cycling and (e) after the first lithiation (discharge to 0.01 V). (f) Top view SEM image of MHSi electrode after 50 cycles.

To investigate the reason of the good cycling stability of MHSi@TiO_2−*x*_-800, the deformation and structural changes during lithiation and delithiation were analyzed by observing the morphologies of MHSi@TiO_2−*x*_-800 after the first lithiation and delithiation processes. As shown in Fig. 6a, the thickness of Si layer in the pristine MHSi@TiO_2−*x*_-800 is 80 nm. After full lithiation (Fig. 6b), the mesoporous structure almost disappears because of the swell of the small silicon crystallites, and the TiO_2−*x*_ shell is maintained without any fracture. The thickness of Si layer increases to 120 nm compared with 80 nm in the pristine state, and the inner void space gets smaller. Therefore, only the expansion to the interior happened, and the outward expansion of Si was confined during the lithiation process due to the press of the rigid TiO_2−*x*_ shell. After the delithiation process, the thickness of Si layer decreases back to 80 nm, and the mesoporous structure can be clearly observed (Fig. 6c), revealing that the structural integrity of the MHSi@TiO_2−*x*_-800 is maintained during the lithiation/delithiation process. Fig. 6d shows that the core–shell morphology of MHSi@TiO_2−*x*_-800 is also well maintained after 50 cycles, indicating robust structural stability during cycling. However, the situation is different for MHSi. As shown in Fig. 6f, the thickness of Si layer increases to approximate 200 nm, and the structure of MHSi cracks. It can be observed that Si expanded outward more easily without the rigid TiO_2−*x*_ coating during the lithiation process. After full delithiation, the MHSi can not get back to the pristine state, and the fracture of Si layer is obvious, indicating bad structural integrity (Fig. 6g). As shown in Fig. 6h, the fracture and pulverization of Si layer in MHSi are very serious after 50 cycles. Fig. 7a and b show the cross-sectional SEM images of MHSi@TiO_2−*x*_-800 electrode before cycling and after the first lithiation process. The volume expansion of MHSi@TiO_2−*x*_-800 electrode is only 12.5%. In comparison, the volume expansion of MHSi electrode is as high as 64% (Fig. 7d and e). The morphological changes of these electrodes after cycling are displayed in Fig. 7c and f. The surface of MHSi@TiO_2−*x*_-800 electrode after 500 cycles is smooth with only some small cracks owing to the rigid TiO_2−*x*_ shell suppressing the outward expansion of Si, resulting in good structural integrity. In contrast, the MHSi undergoes serious electrode surface cracking.

**Fig. 8:**
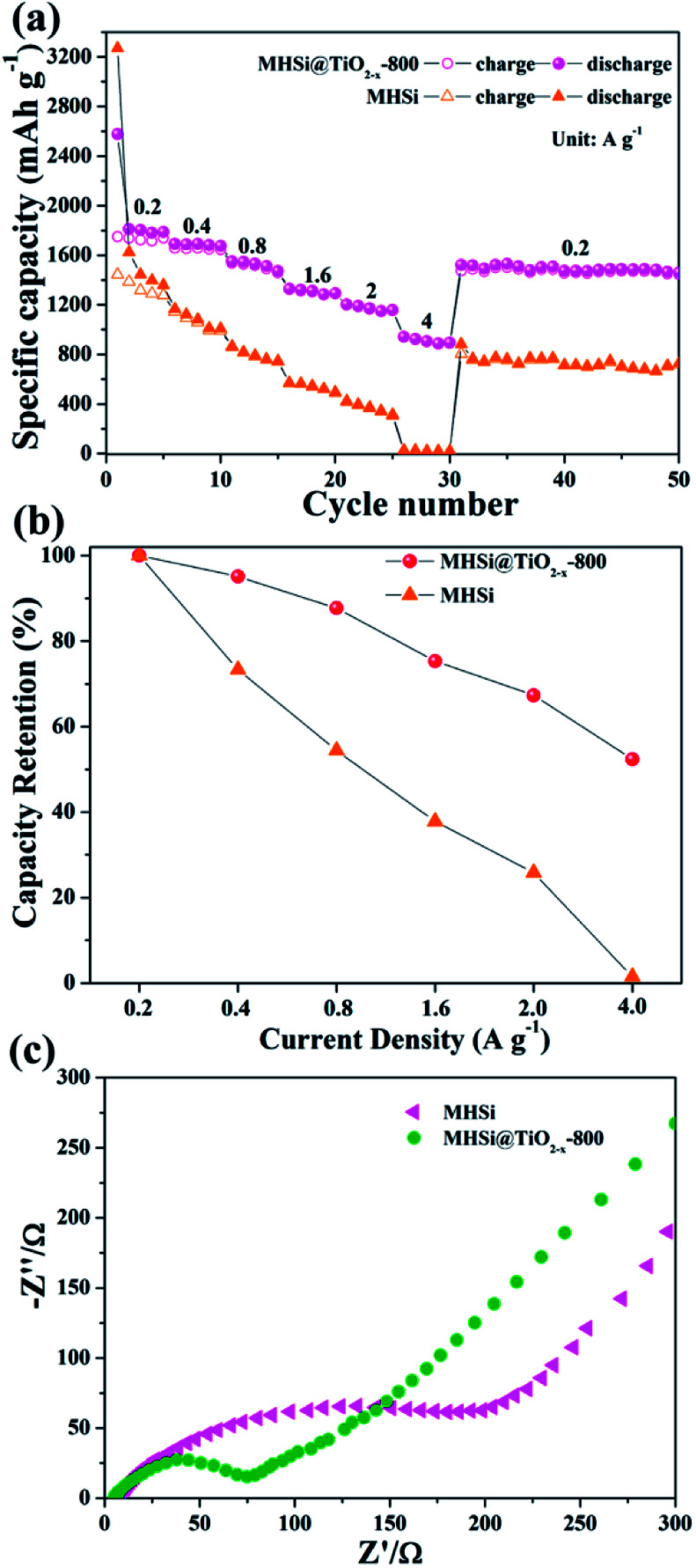
(a) Rate performances of MHSi and MHSi@TiO_2−*x*_-800, (b) capacity retentions of MHSi and MHSi@TiO_2−*x*_-800 at various current densities, and (c) EIS curves of the MHSi and MHSi@TiO_2−*x*_-800 electrodes before cycling.

The MHSi@TiO_2−*x*_-800 composite also exhibits a superior rate capability. As shown in Fig. 8a, at a current density of 0.2 A g^−1^, MHSi@TiO_2−*x*_-800 delivers a high reversible specific capacity of 1750.4 mA h g^−1^. Even when the current density is increased to 4 A g^−1^, a reversible specific capacity of 907.6 mA h g^−1^ is maintained, and the capacity retention is 52.4% (Fig. 8b). A reversible specific capacity of 1530.3 mA h g^−1^ is recovered when the current density is returned to 0.2 A g^−1^ after cycling under high current densities. However, MHSi only delivers a rather low reversible specific capacity of 30.3 mA h g^−1^ at 4 A g^−1^ with a low capacity retention of 1.5%, and a low reversible specific capacity 722.7 mA h g^−1^ is maintained at 0.2 A g^−1^ after cycling under high current densities. Compared with previous studies on Si/TiO_2_ composites (Table S2†), MHSi@TiO_2−*x*_-800 exhibited considerable performance advantages.^37–41,45^ As shown in Fig. 8c, EIS was measured to investigate the reason of the good rate capability of MHSi@TiO_2−*x*_-800. The diameter of the semi-circle for MHSi is much larger than that for MHSi@TiO_2−*x*_-800, indicating faster charge transfer at the electrode/electrolyte interphase owing to the presence of conductive TiO_2−*x*_.

Therefore, the excellent electrochemical performance of MHSi@TiO_2−*x*_-800 is attributed to the features of a mechanically and electrically robust structure. (1) The mesoporous hollow structure provides enough inner void space for the expansion of Si. More importantly, the rigid TiO_2−*x*_ shell suppresses the outward expansion of Si. Under the above combined effect, large volume change is effectively buffered, disintegration of Si is suppressed, and structural integrity is maintained, leading to a high cycling stability. (2) The TiO_2−*x*_ shell not only prevents the direct contact between the electrolyte and Si, but also contributes a slight volume vibration upon lithiation to maintain a stable SEI on the outside surface, leading to a high coulombic efficiency. (3) The oxygen-deficient TiO_2−*x*_ shell provides an enhanced electrical pathway of high conductivity for the transport of electron, and the mesoporous structure is benefit for the diffusion of Li^+^, leading to a superior rate capability.

## Conclusion

In summary, an interface-engineered Si-based anode with a mechanically and electrically robust structure has been synthesized *via* a facile method. The obtained MHSi@TiO_2−*x*_ core−shell nanoparticles feature a mesoporous hollow Si core and a rigid oxygen-deficient TiO_2−*x*_ outer shell. In this architecture, the core–shell structure shows a combined effect to maintain the high interfacial stability and structural integrity. The mesoporous hollow Si core provides enough inner void space for the inward expansion of Si. The TiO_2−*x*_ shell provides a rigid structure with high mechanical stability to suppress the outward expansion of the mesoporous hollow Si nanospheres. Additionally, the TiO_2−*x*_ shell can avoid the direct contact between Si and the electrolyte, and improve the conductivity of composite. As a result, the composite yields a high reversible specific capacity of 1750.4 mA h g^−1^ at 0.2 A g^−1^, an excellent cycling stability of 1303.1 mA h g^−1^ at 2 A g^−1^ with 84.5% capacity retention after 500 cycles, and a high rate capability of 907.6 mA h g^−1^ even at 4 A g^−1^.

## Conflicts of interest

There are no conflicts to declare.

## Supplementary Material

RA-008-C8RA01661E-s001
