# Supplementary material for: Rigid TiO2−x coated mesoporous hollow Si nanospheres with high structure stability for lithium-ion battery anodes
Source: RSC Adv. 2018 Apr 23;8(27):15094–101. doi: 10.1039/c8ra01661e (PMC9080017; doi:10.1039/c8ra01661e)
Supplement: RA-008-C8RA01661E-s001 [file RA-008-C8RA01661E-s001.pdf]

Electronic Supplementary Material (ESI) for RSC Advances.
This journal is © The Royal Society of Chemistry 2018

Electronic Supplementary Information

Rigid TiO_{2-x} Coated Mesoporous Hollow Si Nanospheres with High Structure Stability for Lithium-Ion Battery Anodes

Yongli Yu [a], Gang Li [a], Xu Chen [a], Weiguo Lin [b], Junfeng Rong [b,**] and Wensheng Yang [a,*]

[a] State Key Laboratory of Chemical Resource Engineering, Beijing University of Chemical Technology, Beijing 100029, P.R. China

[b] Research Institute of Petroleum Processing, Sinopec, Beijing 100083, P.R. China

* Corresponding author.

** Corresponding author.

E-mail: yangws@mail.buct.edu.cn (W. Yang), rongjf.ripp@sinopec.com (J. Rong)

1. Characterization of TiO_2 prepared by calcination of the hydrolyzatein of TBOT in air at 800 ^oC

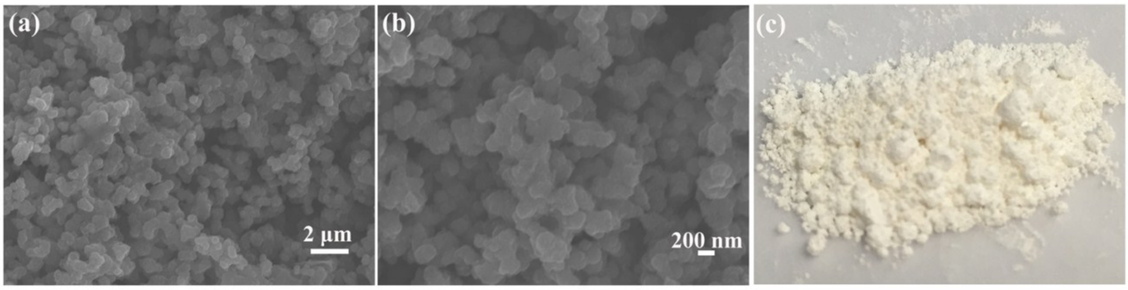

Fig. S1 (a,b) SEM images and (c) digital camera image of TiO_2 prepared by calcination of the hydrolyzatein of TBOT in air at 800 ^oC.

Fig. S1a and b show the SEM images of the TiO_2 nanoparticles prepared by calcination of the hydrolyzatein of TBOT in air at 800 ^oC. The particle size is approximately 300 nm. As shown in Fig. S1c, the TiO_2 shows white color.

2. Characterization of TiO_{2-x} prepared by calcination of the hydrolyzatein of TBOT in Ar flow at 800 ^oC

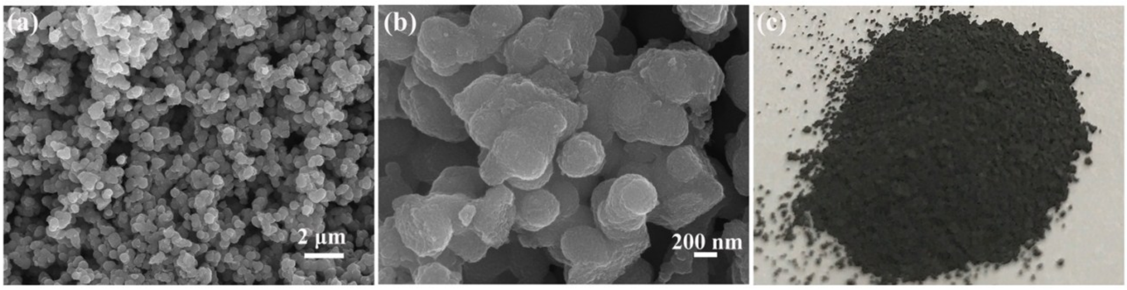

Fig. S2 (a,b) SEM images and (c) digital camera image of TiO_{2-x} prepared by calcination of the hydrolyzatein of TBOT in Ar flow at 800 ^oC.

Fig. S2a and b show the SEM images of the TiO_{2-x} nanoparticles prepared by calcination of the hydrolyzatein of TBOT in Ar flow at 800 ^oC. The particle size is approximately 300 nm, which is same with that of TiO_2 nanoparticles. However, the TiO_{2-x} shows black color. According to the literature (*J. Am. Chem. Soc.*, 2012, **134**, 7600–7603), black TiO_2 usually arises from the oxygen loss in TiO_2, resulting in nonstoichiometric TiO_{2-x}.

3. Characterization of MHSi@TiO_{2-x} prepared by calcination of the intermediate products obtained by the hydrolysis of TBOT on the surface of MHSi in Ar flow at different temperatures

3.1 Morphology characterization

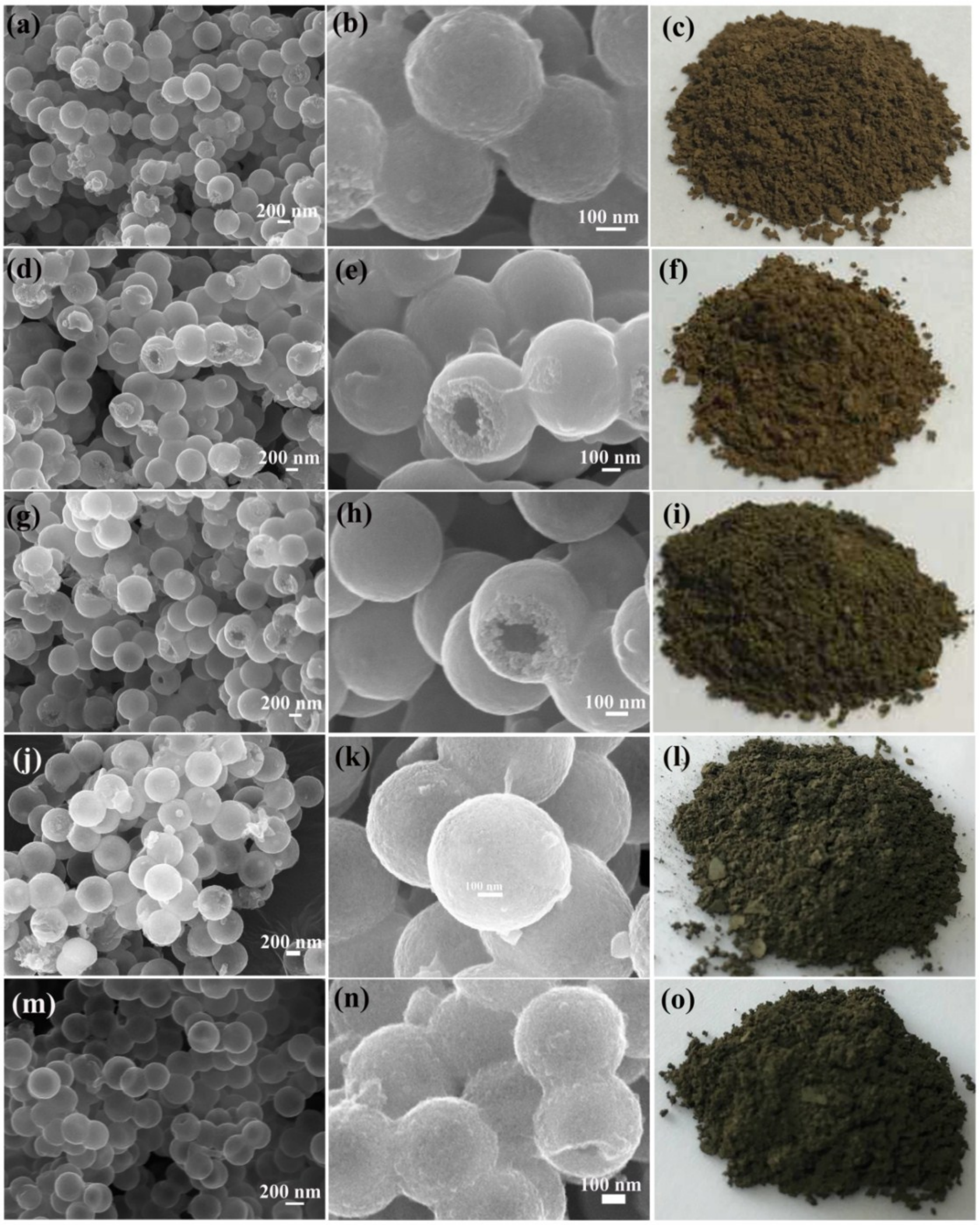

Fig. S3 (a,b) SEM images and (c) digital camera image of MHSi@TiO_{2-x}-500; (d,e) SEM images and (f) digital camera image of MHSi@TiO_{2-x}-600; (g,h) SEM images and (i) digital camera image of MHSi@TiO_{2-x}-700; (j,k) SEM images and (l) digital camera image of MHSi@TiO_{2-x}-800; (m,n)

SEM images and (o) digital camera image of MHSi@TiO_{2-x}-900.

Fig. S3 shows the SEM images of MHSi@TiO_{2-x} prepared by calcination of the products after the hydrolysis of TBOT on the surface of MHSi in Ar flow at different temperatures. All the samples are spherical, and the hollow structures, observed from the broken spheres, are still maintained. The surfaces of the samples are smooth except MHSi@TiO_{2-x}-900 (Fig. S3n), which is attributed to the particle aggregation of TiO_{2-x} under high-temperature calcination of 900 °C. Many reports have discussed coloration of TiO_2 in relation with the electronic structure (*Nat. Chem.*, 2011, **3**, 296–300; *J. Am. Chem. Soc.*, 2012, **134**, 7600–7603; *J. Phys. Soc. Jpn.*, 2004, **73**, 703–710). As shown in Fig. S3c, f, i, l, and o, the coloration of these samples gets gradually dark-green, implying the presence of nonstoichiometric TiO_{2-x} in our samples.

3.2 Electrochemical cyclic performance test

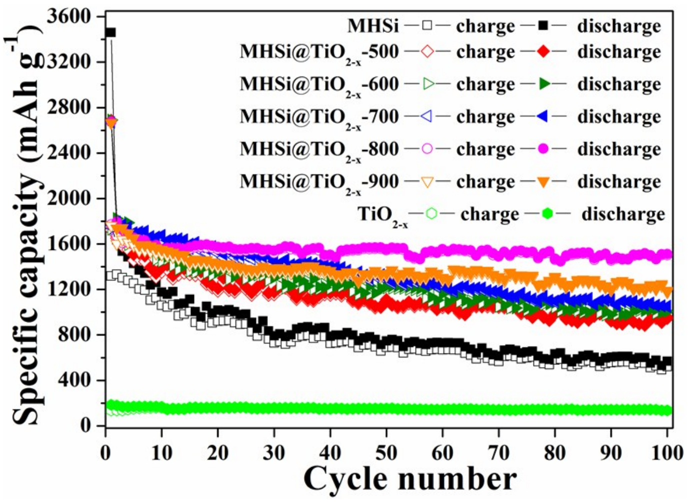

Fig. S4 Electrochemical cyclic performances of MHSi, MHSi@TiO_{2-x}-500, MHSi@TiO_{2-x}-600, MHSi@TiO_{2-x}-700, MHSi@TiO_{2-x}-800, and MHSi@TiO_{2-x}-900 at 0.4 A g^{-1}.

Table S1. Cyclic performance comparison among MHSi, MHSi@TiO_{2-x}-500, MHSi@TiO_{2-x}-600, MHSi@TiO_{2-x}-700, MHSi@TiO_{2-x}-800, and MHSi@TiO_{2-x}-900

Sample	Initial reversible specific capacity (mAh g^{-1})	Reversible specific capacity after 100 cycles (mAh g^{-1})	Capacity retention
MHSi	1317.7	521.9	39.6%
MHSi@TiO_{2-x}-500	1723.1	945.4	54.8%
MHSi@TiO_{2-x}-600	1730.2	974.1	56.3%
MHSi@TiO_{2-x}-700	1751.4	1071.8	61.2%
MHSi@TiO_{2-x}-800	1770.6	1502.1	84.8%
MHSi@TiO_{2-x}-900	1762.4	1238.9	70.3%

As shown in Fig. S4, the electrochemical cyclic performances of MHSi, MHSi@TiO_{2-x}-500, MHSi@TiO_{2-x}-600, MHSi@TiO_{2-x}-700, MHSi@TiO_{2-x}-800, and MHSi@TiO_{2-x}-900 as anode materials for LIBs were preliminarily evaluated. MHSi shows a higher initial discharge specific capacity, but the initial coulombic efficiency is only 40.1%. While, the initial coulombic efficiency of MHSi@TiO_{2-x}-500, MHSi@TiO_{2-x}-600, MHSi@TiO_{2-x}-700, MHSi@TiO_{2-x}-800, and MHSi@TiO_{2-x}-900 are 64.1%, 64.3%, 65.6%, 66.2% and 66.1%, respectively, indicating that TiO_{2-x} coating is benefit for the improvement of the initial coulombic efficiency. The initial reversible specific capacities, the reversible specific capacities after 100 cycles, and the corresponding capacity retentions of MHSi and the samples calcined at different temperatures are summarized in Table S1. MHSi@TiO_{2-x}-800 exhibits the highest initial reversible specific capacity, but it is basically the same with the initial reversible specific capacities of the samples calcined at other temperatures. This is because the contents of Si in these samples are almost the same. Besides, the chemical components of TiO_{2-x} in these samples may be different, but the reversible specific capacity of TiO_{2-x} is only 140 mA h g^{-1} (Fig. S4), and the contents of TiO_{2-x} in these samples are

not high (See Fig. S6 and the corresponding discussion), so the contribution of TiO_{2-x} to the total specific capacity of the composite is very little. Therefore, there are little differences in the initial reversible specific capacities of these samples. After 100 cycles, the capacity retentions of these samples gradually increase with the increase of the calcination temperature, except MHSi@TiO_{2-x}-900. According to Fig. S3, these samples show different colors, which are in relation with the electronic structure of TiO_{2-x}, implying different electrical conductivities. As shown in Fig. S5, the electrical conductivities of MHSi@TiO_{2-x}-500, MHSi@TiO_{2-x}-600, MHSi@TiO_{2-x}-700, MHSi@TiO_{2-x}-800, and MHSi@TiO_{2-x}-900 were evaluated. The semicircles in the Nyquist plots are in relation with the charge transfer resistance. The diameters of the semicircles of these samples (MHSi@TiO_{2-x}-500, MHSi@TiO_{2-x}-600, MHSi@TiO_{2-x}-700, MHSi@TiO_{2-x}-800) decrease with the increase of the calcination temperature, indicating that the electrical conductivities of these samples increase. The diameter of the semicircle of MHSi@TiO_{2-x}-800 is very close to that of MHSi@TiO_{2-x}-900, indicating that little increase in the electrical conductivity is obtained. The improvement of the electrical conductivity facilitates the formation of a stable conductive network inside the samples during cycling, promoting the utilization rate of Si. Additionally, with the increase of the calcination temperature, the strength of TiO_{2-x} may increase (Although the strength of TiO_{2-x} in the composite is not easily measured), which is benefit for the improvement of structural stability. However, as shown in Fig. S3n, particle aggregation of TiO_{2-x} exists when the calcination temperature increases to 900 ^{o}C, leading to the inhomogeneous coating of TiO_{2-x}. The inhomogeneous coating layer of TiO_{2-x} may fracture during cycling, resulting in bad structural stability of the composite. Therefore, MHSi@TiO_{2-x}-800 shows the highest capacity retention.

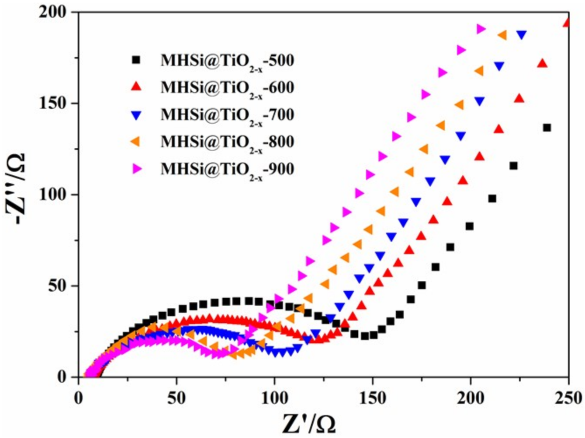

Fig. S5 EIS curves of the MHSi@TiO_{2-x}-500, MHSi@TiO_{2-x}-600, MHSi@TiO_{2-x}-700, MHSi@TiO_{2-x}-800, and MHSi@TiO_{2-x}-900 electrodes before cycling.

4. EDS elemental analysis of MHSi@TiO_{2-x}-800

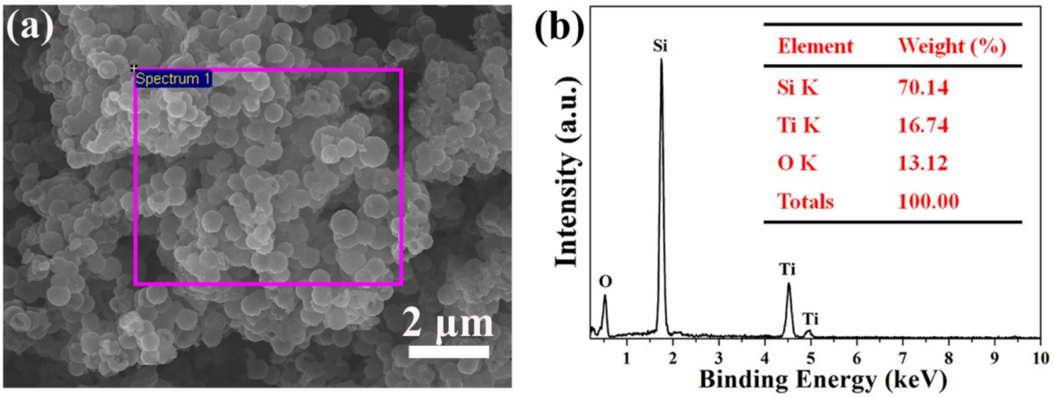

Fig. S6 (a) SEM image and (b) the corresponding EDS spectrum of MHSi@TiO_{2-x}-800 (the embedded table shows the elemental contents of Si, Ti and O).

As shown in Fig. S6, the EDS spectrum of MHSi@TiO_{2-x}-800 shows the characteristic peaks of Si, Ti and O. The embedded table shows that the elemental content of Ti is 16.74%, and that of O is 13.12%. According to the chemical components of $TiO_{1.939}$, which is obtained from the analysis of the X-ray photoelectron spectroscopy of MHSi@TiO_{2-x}-800, the corresponding elemental content of O should be 10.82%. The difference between the above two elemental contents of O is ascribed to the partial oxidation of Si. So the content of TiO_{2-x} in MHSi@TiO_{2-x}-800 is 27.6%.

5. BET characterization of MHSi@TiO_{2-x}-800 and MHSi

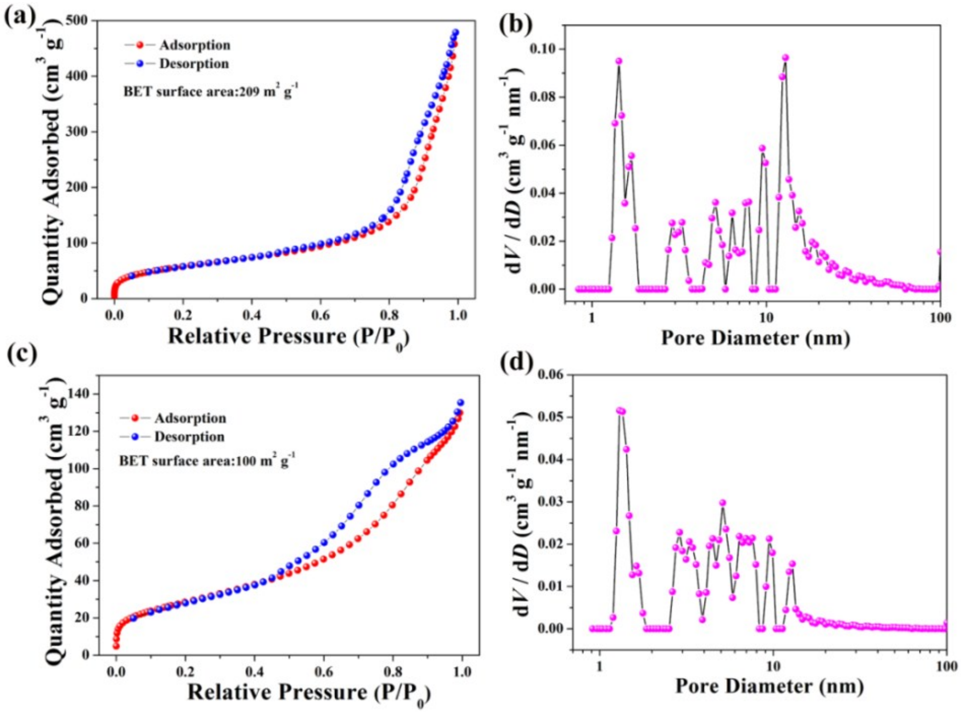

Fig. S7 (a) Nitrogen sorption isotherms and (b) pore size distribution of MHSi; (c) Nitrogen sorption isotherms and (d) pore size distribution of MHSi@TiO_{2-x}-800.

As shown in Fig. S7a and c, nitrogen sorption isotherms of both MHSi and MHSi@TiO_{2-x}-800 exhibit typical *IV* curves with distinct H1 hysteresis loops. The Brunauer−Emmett−Teller (BET) surface area of the MHSi is 209 m^2 g^{-1}. After TiO_{2-x} coating, the BET surface area of the MHSi@TiO_{2-x}-800 gets smaller to 100 m^2 g^{-1}, which is ascribed to the decrease of some mesopores with pore diameter of 10 nm (Fig. S7b and d). The reduction of the specific surface area is beneficial to reduce the growth of SEI film. The mesopores with pore diameter of 1~2 nm are still maintained, which will not prevent the infiltration of electrolyte.

6. Electrochemical performance comparison between MHSi@TiO_{2-x}-800 in this work and other Si/TiO_2 composites reported in previous studies

Table S2. Performance comparison between MHSi@TiO_{2-x}-800 in this work and other Si/TiO_2 composites reported in literatures.

Materials	Reversible specific capacity (mAh g^{-1})	Rate performance (mAh g^{-1})	Capacity retention	Ref.*
MHSi@TiO_{2-x}-800	1750.4 at 0.2 A g^{-1}	907 at 4 A g^{-1}	84.5% after 500 cycles	This work
Void-involved Si@TiO_2 nanospheres	1671 at 0.1 A g^{-1}	710 at 1 A g^{-1}	48.1% after 100 cycles	[37]
Si@mesoporous C@TiO_2 nanoparticles	2775 at 0.14 A g^{-1}	697 at 11.2 A g^{-1}	36.4% after 700 cycles	[38]
Core-shell Si@TiO_2 nanoparticles	1580.3 at 0.4 A g^{-1}	/	55.3% after 50 cycles	[39]
pineapple-structured Si/TiO_2 composite	1200 at 0.4 A g^{-1}	300 at 2 A g^{-1}	58.3% after 50 cycles	[40]
Yolk-shell structured Si@TiO_2 composite	1562 at 0.4 A g^{-1}	990 at 2 A g^{-1}	63.4% after 1500 cycles	[41]
Core-shell Si@TiO_{2-x}/C microfiber	1000 at 0.2 A g^{-1}	939 at 12 A g^{-1}	90% after 50 cycles	[42]

Ref.* refers to the reference number in the main paper.
